# Humane Endpoints in Swiss Webster Mice Infected with *Toxoplasma gondii* RH Strain

**DOI:** 10.3390/ani14091326

**Published:** 2024-04-29

**Authors:** Igor Falco Arruda, Maria Regina Reis Amendoeira, Thamires Francisco Bonifácio, Clarissa Nascimento da Silveira Raso, Hyago da Silva Medeiros Elidio, Jhônata Willy Rocha Coelho, Luiz Cesar Cavalcanti Pereira da Silva, Isabele Barbieri dos Santos

**Affiliations:** 1 Toxoplasmosis and Other Protozoan Diseases, Oswaldo Cruz Institute, Fiocruz, Manguinhos, Rio de Janeiro 21040-900, Brazil; amendoei@ioc.fiocruz.br (M.R.R.A.); thamires.bonifacio@ioc.fiocruz.br (T.F.B.); clarissaraso@aluno.fiocruz.br (C.N.d.S.R.); 2 Center for Animal Experimentation, Oswaldo Cruz Institute, Fiocruz, Manguinhos, Rio de Janeiro 21040-900, Brazil; hyago.elidio@ioc.fiocruz.br (H.d.S.M.E.); jhonata.coelho@ioc.fiocruz.br (J.W.R.C.); luiz.cavalcanti@fiocruz.br (L.C.C.P.d.S.); isabele.santos@ioc.fiocruz.br (I.B.d.S.)

**Keywords:** humane endpoints, refinement, toxoplasmosis, murine model

## Abstract

**Simple Summary:**

Toxoplasmosis is a globally distributed zoonosis caused by *Toxoplasma gondii*. This protozoan is capable of infecting mammals and birds and can cause significant impacts on human and animal health. Much of what is known about the parasite’s biology, the immune mechanisms involved in toxoplasmosis, and the development of diagnostic tests for this protozoan disease is due to the maintenance of the parasite in laboratory mice. However, the increasing debate on the use of biomodels in scientific research has brought about the need to implement the principles of the 3Rs in laboratory protocols, such as experimental infections with *T. gondii* in murine models. Therefore, this study aimed to establish the humane endpoints for experimental infection with the *T. gondii* RH strain in Swiss Webster mice. The results presented here seek to disseminate the method we have proposed for the humane finalization of these animals in experimental protocols using this highly virulent strain as a method of refinement that seeks to enhance animal welfare.

**Abstract:**

The highly virulent *Toxoplasma gondii* RH strain is maintained through successive passages in mice, but there is still a lack of studies that refine these procedures from a 3Rs perspective, where humanitarian ideals aim to minimize the stress, pain, or suffering of the animals used in the research without the loss of results. The aim of this study was to establish humane endpoints in Swiss Webster mice inoculated with the *T. gondii* RH strain. A total of 52 mice were infected with 5 × 10^6^ tachyzoites/mL and monitored for periods of up to 5 days. The parameters body weight; hair condition; higher than normal body temperature; hypothermia; respiratory function; pain; soft stools or diarrhea; bloody diarrhea; tense, nervous, or in distress during handling; and ascites were recorded daily in score tables. The results showed that prominent piloerection, respiratory function, pain parameters, and ascites are important clinical signs to be used as a cut-off point for implementing euthanasia. The application of this refinement method helped to avoid animal suffering and pain without compromising the number of parasites recovered. We therefore suggest adopting these parameters in research protocols that require the maintenance of the *T. gondii* RH strain in murine models to avoid and reduce animal suffering.

## 1. Introduction

Toxoplasmosis is a zoonosis caused by the protozoan *Toxoplasma gondii*, which has felids as definitive hosts and birds and mammals, including humans, as intermediate hosts [1,2]. Immunocompromised individuals and pregnant women are risk groups for human toxoplasmosis due to the possibility of more severe manifestations with serious sequelae [3,4,5,6]. Global estimates of *T. gondii* infection indicate that around a third of the world’s population is infected [6]. In Brazil, the prevalence varies between 50% and 80% and can reach 100% in certain locations, depending on the habits, customs, climate, and socioeconomic conditions of each region [7,8,9]. Brazil also has one of the highest incidence rates of congenital toxoplasmosis in the world, around 6 in every 10,000 live births, as well as significant rates of acquired ocular toxoplasmosis, affecting around 17.7% of the population [10,11].

The *T. gondii* population structure is complex, consisting of clonal strains (genotypes I, II, and III) and atypical strains [12,13]. Among the clonal strains, the *T. gondii* RH strain is widely used by different research groups around the world in seroepidemiological, pharmacological, and cell biology studies, among others [14,15,16,17,18,19]. Originally isolated in mice by Sabin [20], from a fatal case of infant toxoplasmic encephalitis, the RH strain is classified as genotype I, showing high virulence and a low lethal dose in a murine model [21].

Mice are a natural intermediate host of *T. gondii* and, as such, are excellent models for the isolation of this protozoan from biological and environmental samples, identifying differences in pathogenesis between parasite strains, and studying immune mechanisms involved in infection control [22,23,24]. Despite the standardization of protocols for *T. gondii* RH strain maintenance in mice, there is still a lack of studies that refine these procedures from a 3Rs perspective (replacement, reduction, and refinement). Howe and Sibley [25] identified genetic variations between seven strains of the RH strain using PCR-RFLP. It is possible that, after 80 years since its isolation and successive passages in animals or cell cultures, these genetic variations may determine different phenotypes of clinical alterations in the murine model.

The humane endpoint is the moment at which the pain, discomfort, or stress of an animal used in a teaching or research activity is avoided, ended, minimized, or reduced, allowing the scientific objectives of the research protocol to be achieved while minimizing animal suffering. Drawing up the appropriate humane endpoints for each species and each procedure used in an experimental protocol is fundamental to the ethical suitability of the research project. This is an obligatory topic that must be thoroughly prepared by researchers. It is important to emphasize that the main objective of determining the humane endpoints is to prevent animals from reaching a moribund state, i.e., to determine a point before welfare is compromised so that action can be taken to meet the criteria of weight, temperature, motor activity, posture, hair condition, hygiene, appetite, aggression, facial expression, vocalization, appearance, and response to handling, which should be assessed to determine the right time to euthanize the animal [26,27].

In view of the above, given the susceptibility of mice to *T. gondii* infection, the aim of this study was to determine the humane endpoints of Swiss Webster mice infected with *T. gondii,* the RH strain, using scoring tables to help quantify the impairment of animal welfare by assessing clinical and behavioral parameters.

The hypothesis of this study was to demonstrate whether the implementation of humane end points contributed as a refinement method, avoiding unnecessary suffering and pain for the animals and allowing the *T. gondii* RH strain to be maintained in a murine model without compromising the quantity of parasites recovered at each passage in the animals.

## 2. Materials and Methods

### 2.1. Animals 

This study included 60 Swiss Webster mice aged between 4 and 5 weeks, divided into two groups: group 1 (infected), composed of 52 animals infected with *T. gondii* RH strain, and group 2 (control), composed of 8 uninfected mice. This evaluation included male and female Swiss Webster mice, aged between 4 and 5 weeks, requested by researchers using the animal facility, whose research protocols had been approved by the IOC/Fiocruz Ethics Committee on the Use of Animals, and included experimental infection with *T. gondii* RH strain. Mice that showed any behavioral and/or physical alterations when they arrived at the animal facility and that negatively influenced their well-being were excluded from follow-up.

The animals evaluated in this study were selected for convenience, as they would already be used in other protocols conducted in the animal facility that included experimental infection with the *T. gondii* RH strain for another research study. Therefore, it is worth emphasizing that the humane endpoints evaluation proposed here was designed in such a way as not to require the infection of new animals, taking advantage of those already infected for other scientific purposes. For the follow-up, an expected frequency of death in the first three days of infection of 25% and a significance level of 95% were considered. This estimate was based on our group’s experience acquired during the routine maintenance of the *T. gondii* RH strain in Swiss Webster mice for other studies. Finally, it would be necessary to include a minimum of 54 animals. 

The animals in each cage were individually marked with non-toxic, odorless paint on the front paw, back paw, tail, and one unmarked animal. The unmarked animal was part of the experiment. The absence of marking was used as a way of differentiating this animal from the other 3 marked animals in the cage for the clinical evaluation. These animals were kept in microisolators (Alesco^®^, Monte Mor, Brazil), equipped with a controlled ventilation system (15 air changes per hour), with environmental enrichment items to stimulate natural behavior (paper, hay, igloo, hydrophobic cotton, and PVC pipe), with a 12-h light/dark regime, a room temperature of 21 + 2 °C, and the supply of water and food suitable for mice, treated with autoclaving, “ad libitum”, in the laboratory animal facility of the Carlos Chagas Pavilion, Center for Animal Experimentation (CEA), Oswaldo Cruz Institute (IOC), Oswaldo Cruz Foundation (Fiocruz).

### 2.2. Experimental Infection of Mice with Tachyzoites of T. gondii RH Strain

The animals were physically restrained for the experimental infection. For restraint, the animals were suspended by the base of their tails and supported on the lid of the cage so that they could hold on. They were then pressed lightly onto the surface of the lid and held by the skin of the dorsocervical region, between the index finger and thumb, clamping their tails between the other fingers and the palm of the hand, to completely limit their movements. After immobilization, the animals were inoculated intraperitoneally in the lower left quadrant of the abdomen with approximately 200 µL of a suspension of 5 × 10^6^ tachyzoites/mL of *Toxoplasma gondii* RH strain [24]. This concentration is standardized for maintaining and obtaining the parasite in the Toxoplasmosis and other Protozoan Diseases Laboratory, currently Protozoology Laboratory.

### 2.3. Monitoring the Clinical and Behavioral Parameters of Infected Mice

Eight parameters were established for the daily clinical/behavioral assessment of the animals, each parameter with its own sub-items and each worth a score, as follows: body weight (5–10% weight loss: 1; 11–15% weight loss: 2; 16–20% weight loss: 3; >20% weight loss: euthanasia); hair condition (slight piloerection: 2; prominent piloerection: 3); change in body temperature (up to 2 °C above normal: 2; up to 3 °C above normal: 3; hypothermia <35 °C: euthanasia); respiratory function (tachypnea: 2; dyspnea: 3); pain parameters (moderately present orbital tightening: 1; intense present orbital tightening: euthanasia; eye discharge: 1; chest breathing: 2; arched posture: 3); environment (soft stools or diarrhea: 1; bloody diarrhea: euthanasia); behavior (tense or nervous during handling: 1; in noticeable distress during handling such as tremors, vocalizations, aggressiveness: 3), specific indicators of infection (ascites: euthanasia) (Table 1). The maximum score in the table would be 23 points if the animal had all the symptoms described above at the highest intensity. When the animal’s score reached 4, the animal was observed twice a day (morning and afternoon), and if the score reached 6, the animal was euthanized to prevent it from becoming moribund. Furthermore, even if score 6 was not reached but the animal showed symptoms of extreme suffering such as >20% weight loss, hypothermia <35 °C, bloody diarrhea, and ascites, it was euthanized. The temperature was measured with a surface infrared thermometer (DM300 Zoss^®^, Taipei, Taiwan) aimed at the xiphoid process at a distance of approximately 0.5 cm from the animal’s body. This thermometer operates in a range from −22° to 43° C. Body weight was measured on a balance (Electronic Kitchen Scale SF-400^®^, Taipei, Taiwan) with a precision of ±0.1 g. This monitoring took place over 5 days, with day zero being the day of infection and the fourth day post-infection (DPI) being the maximum survival time of the animals. The scoring system used in the current study to assess mice was adapted from those developed to assess the clinical score of other experimental parasitic infections [28,29].

### 2.4. Quantification of T. gondii Tachyzoites after Experimental Infection

Infected mice that reached a score of 6 were euthanized intramuscularly with a combination of 300 mg/kg 10% ketamine hydrochloride and 30 mg/kg 2% xylazine hydrochloride [26]. Death was confirmed by the absence of respiratory movements and a heartbeat using a stethoscope. To obtain the tachyzoites, an intraperitoneal lavage was carried out with 10 mL of refrigerated (4 to 8 °C) sterile 1% phosphate-buffered saline (PBS) (P4417 code, Sigma-Aldrich^®^, St. Louis, CA, USA). The washings were transferred to a single 50 mL conical-bottom polypropylene tube and centrifuged at 1000× *g* for 10 min to concentrate the parasites. The supernatant was then discarded, and the sediment was resuspended in sterile 1% PBS. From the concentrate, the parasites were counted in a Neubauer chamber to adjust the concentration of tachyzoites for a new inoculum at room temperature (23 to 25 °C). A new group of four mice was infected intraperitoneally with the same parasite concentration (5 × 10^6^ tachyzoites/mL) to continue monitoring.

### 2.5. Statistical Analysis

The sample was calculated using the virtual statistical calculator of the University of São Paulo/Brazil (http://estatistica.bauru.usp.br/calculoamostral/ta_diferenca_proporcao.php) accessed on 14 March 2024. For this purpose, an error of 5% was considered; the power of the test was 80%; and the frequencies of death within three days of infection were 0% and 25% in uninfected and infected mice, respectively. From the score data, the first day on which the mouse received a score was identified. Subsequently, the number of days between the moment when a given score was first observed and the death of the animal (by observation of the dead body or euthanasia) was calculated. Survival times were estimated from the first day after infection until the observed death or euthanasia of each animal.

Body weight, temperature, and pre-established scores for each clinical/behavioral parameter were recorded daily, starting on the day of inoculation, in Microsoft Excel^®^ version 2016 (Washington DC, USA) sheets. In addition, the concentrations of tachyzoites recovered after euthanasia were recorded on the same sheets. A statistical analysis of the clinical and behavioral parameters evaluated was performed with data collected on the 2nd and 3rd days of DPI. It is worth noting that, on the 1st DPI, euthanasia was not implemented as the score of the monitored animals did not reach the value of 6, and on the 4th DPI, all previously surviving animals were euthanized. Initially, to verify the association between continuous variables (body weight and temperature) and the implementation of euthanasia, a one-way ANOVA was performed. Additionally, normality analysis of the continuous parameters was conducted by the Shapiro-Wilk normality test. To evaluate the association between the nominal categorical variables, the Fischer Exact Test was performed. Then, the recorded score values were included in a multiple linear regression model to evaluate the association between the scores and the evaluated clinical and behavioral parameters. Finally, the association between the mean concentration of recovered tachyzoites and the day of euthanasia implementation was also estimated by a one-way ANOVA. *p*-values ≤ 0.05 were considered significant. Statistical analyses were performed using Jamovi version 2.3.28 (Sydney, Australia).

### 2.6. Ethics

The procedures adopted in the study followed international welfare standards and were carried out in accordance with Brazilian legislation licensed under number L-029/2018 by the Oswaldo Cruz Institute’s Animal Use Ethics Committee (CEUA/IOC/Fiocruz).

## 3. Results

From a global data collection in which all the clinical and behavioral parameters described in Table 1 were evaluated for each animal daily, we obtained the following results.

### 3.1. Group 1

Slight and noticeable piloerection was observed in 98% (n = 51) of the animals. Survival time (time between day 0 and day 5 that the animal remained alive) due to death or euthanasia was 3 days in 32% (n = 16), 4 days in 48% (n = 25), and 5 days in 18% (n = 9). The time between the first symptoms appearing and euthanasia was 2 days in 56% (n = 28), 3 days in 36% (n = 18), and 4 days in 8% (n = 4). The first symptom that appeared in 80% (n = 40) of the animals was slight piloerection, and in 20% (n = 10) of the animals, slight piloerection was observed along with 5 to 10% loss of body weight. Weight loss was between 5 and 10% in 60% (n = 30) of the animals, 11 to 15% in 18% (n = 9), 16 to 20% in 4% (n = 2), and more than 20% in one animal (2%), when the last weight was collected. Dyspnea was observed in 24% (n = 12) of the animals, moderately present orbital tightening in 20% (n = 10), chest breathing in 36% (n = 18), arched posture in 20% (n = 10), and ascites in 66% (n = 33). During the 5 days, the temperature remained within the normal range for all the animals, with the lowest being 35.7 °C and the highest being 38 °C. The clinical alterations of intense present orbital tightening, tachypnea, eye discharge, soft stools or diarrhea, bloody diarrhea, changes in tense or nervous behavior during handling, tremors, vocalizations, and aggressiveness were not observed in any animal.

The mice were euthanized when they reached score 6 and/or when the animal presented ascites, which occurred between the second, third, and fourth DPI (mean: 3.7 ± 0.46 days). Two animals that had noticeable piloerection and weight loss of between 5% and 10% (total score 4) were found dead on the second DPI. The remaining 50 animals were euthanized based on the clinical and behavioral parameters described in the methodology of this study, with 18% (n = 9) euthanized on the fourth DPI, 48% (n = 25) on the third DPI, and 32% (n = 16) on the second DPI.

The mean concentration of tachyzoites recovered from the euthanized animals according to the proposed clinical score was 2.8 × 10^7^ tachyzoites/mL, ranging from 1.1 × 10^7^ to 7 × 10^7^ tachyzoites/mL (Table 2). When comparing the means values of tachyzoites recovered from the mice euthanized on the days of second, third, and fourth DPI, no significant difference was observed between the number of parasites recovered on these days (*p* = 0.2149).

Except for the weight recorded on the second DPI (*p* = 0.214), the other continuous parameters recorded on the second and third DPI did not show a normal distribution (second DPI: temperature and score; *p* < 001/third DPI: weight; *p* = 0.048; temperature and score; *p* < 001). The initial bivariate analysis revealed a significant association between the implementation of euthanasia and the following clinical and behavioral parameters on the second DPI: hair condition, chest breathing, and ascites (*p*-value ≤ 0.05). On the third DPI, the implementation of euthanasia was associated with body weight and ascites (*p*-value ≤ 0.05) (Table 3 and Table 4). The final multiple linear regression model indicated the contribution of hair condition, respiratory function, chest breathing, and ascites to the increase in scores recorded on the second DPI (*p*-value ≤ 0.05; adjusted R^2^: 0.95). On the third DPI, body weight, hair condition, chest breathing, and ascites were predictive parameters of euthanasia responsible for the increase in recorded scores (*p*-value ≤ 0.05; adjusted R^2^: 0.69) (Table 5). 

### 3.2. Group 2

None of the animals showed any clinical or behavioral changes. During the five monitoring days, the temperature remained within the normal range in all the animals, with 36.0 °C being the lowest and 37.8 °C the highest.

## 4. Discussion

Based on the results observed in this study, it was possible to establish clinical and behavioral parameters for the implementation of humane endpoints in Swiss Webster mice infected with the *T. gondii* RH strain. The *T. gondii* RH strain has high virulence and lethality in all strains of laboratory mice [30,31]. Type I clonal strains, such as the RH strain, or atypical strains with a virulent phenotype of *T. gondii,* determine an acute infection profile in mice that progresses rapidly due to the expansion and dissemination of parasites to all organs of the body, leading to cytokine shock and death within the first 10–12 days [32,33,34]. Given this fulminant and fatal prognosis, attempts to replace, reduce, or refine the protocols that use the murine model for the maintenance of *T. gondii* RH strain are necessary. However, the use of mice in certain protocols (for example, attempts to isolate and evaluate the virulence of parasite isolates) is still necessary. Thus, methods that refine the use of these animals, prioritizing their well-being, are indispensable, such as the implementation of the humane endpoints proposed in this study.

The evaluation of the parameters proposed here was carried out using a scoring table. The advantages of a scoring system based on the quantification of clinical signs (scores) include careful observation of the animals, especially during the critical period of the experimental protocols. Score tables can reveal patterns of deterioration or recovery over time, as well as encourage researchers to observe and recognize normal and abnormal behavior in their respective biomodels in response to experimental parameters [34]. For this reason, in this study we established eight parameters for the daily clinical/behavioral evaluation of the animals, and in the end, only five of these were relevant for this evaluation.

Thus, we defined the clinical signs of infection with the *T. gondii* RH strain as the hair condition, respiratory function, pain parameters, and ascites.

Hair condition (prominent piloerection), respiratory function (dyspneia), chest breathing, body weight, and ascites were identified as endpoints for infection with *T. gondii* RH strain in Swiss Webster mice (Table 5). These signs and symptoms were also described by Sabin [20] when this strain was isolated and, more recently, by Sudan et al. [35] in Swiss Webster mice infected with the RH strain. In addition, these same parameters have also been described in laboratory mice infected with atypical *T. gondii* strains of virulent phenotype isolated from domestic and wild animals [36,37,38]. In this way, the humane endpoints identified in this study can also be implemented in research protocols that use not only the RH strain and others of clonal genotype type I but also atypical strains of virulent phenotype, in line with good animal experimentation practices.

Among these clinical signs, prominent piloerection stood out as the parameter that was more predictive of death than the others. Euthanizing animals with notorious piloerection would have minimized the pain and anguish of 12 mice that were euthanized due to ascites and 2 that were found dead on the second day of infection. In addition, noticeable piloerection was observed between the second and third days post-infection, at which time an optimum mean concentration of tachyzoites was observed for the maintenance of *T. gondii* RH strain. Considering that the number of tachyzoites did not vary significantly, the results show that the identification of notorious piloerection between the second and third days of infection is already predictive of the implementation of the humanitarian finalization of experimental protocols using the *T. gondii* RH strain in Swiss Webster mice. 

Physiological parameters such as hematology, biochemistry, and urinalysis were not evaluated. This was a limitation of our study. We did not assess these parameters because the laboratory that performs these tests has a deadline of 2 to 3 days to publish the results, and to include these assessments in our score, we would need daily results due to the rapid course of the *T. gondii* RH strain infection.

In this study, we carefully observed the animals’ physiological, behavioral, and pain parameters daily. However, due to logistical limitations, we did not film the animals, which would have made it easier to monitor their behavior and pain assessment parameters.

The use of morbidity as a substitute for mortality requires the identification of criteria that can be used to define morbidity and verify its applicability as a substitute for death in endpoint studies [39,40]. Clinical signs can provide a more objective judgment of animal pain and distress. According to Morton [35], researchers must recognize these natural behavioral and physical parameters of animals and develop skills for clinical observation of biomodels and the description of specific symptoms of the infection carried out in their experimental protocols. Therefore, we believe that a simple and objective methodology for determining the humane endpoints and assessing animal welfare is essential, as is the commitment of researchers to carry out rigorous monitoring of the animals to identify the moment of intervention and the humane endpoints. In situations of invasive interventions or acute and fulminant experimental infections, the frequency of monitoring should be increased, and it is recommended that animals be assessed twice a day, in the morning and late afternoon [28,29].

The definition of the humane endpoints identified in this study was possible based on the scores established in the score table. Therefore, the score tables should be simple and objective so that they make it easier to assess the animals and have results that are consistent with reality. They should contain general clinical and behavioral parameters for evaluating the animals (weight, temperature, motor activity, posture, hair condition, hygiene, appetite, aggression, facial expression, vocalization, appearance, and response to handling), as well as specific parameters related to the outcomes to which the animals will be subjected in the experimental protocol (e.g., appearance of the surgical scar, tumor size, expected side effects of the drugs administered to the animals, symptoms of the disease). The gradation value of the parameters should not be complex or extremely detailed so as not to make it difficult to assess the animal [28,29]. 

The clinical and behavioral parameters of the mice infected with *T. gondii* RH strain were evaluated with simple scores ranging from 1 to 3 to facilitate the observation of clinical signs. In this way, we reduced the subjectivity of the evaluation of clinical and behavioral parameters, making it possible to translate what was observed in the animals into evidence that could be documented and scored. It is important to note that observers should be trained in recognizing and scoring clinical and behavioral signs so that they can carry out this observation in the most similar way possible.

## 5. Conclusions 

The results of this study suggest that the observation-based scoring system is an effective tool for mitigating pain and suffering in mice through preventive euthanasia, and efforts must be made to make the assessment of behavioral features as uniform and consistent as possible so that the evaluation criterion is repeatable and reproducible. Identifying parameters of pain, respiratory function, and, in particular, prominent piloerection should be used accurately as humane endpoints for euthanizing mice before they become moribund. Assigning scores quantifying a series of clinical signs provided an effective mechanism for documenting clinical progression and facilitated the decision on when to euthanize the animal as the toxoplasmic infection progressed. The humane endpoints implemented in this study indicate that the application of this refinement method helped to avoid unnecessary suffering and pain for the animals but also allowed the *T. gondii* RH strain to be maintained in a murine model without compromising the quantity of parasites recovered at each passage in the animals.

## Figures and Tables

**Table 1:** Parameters for daily clinical/behavioral assessment of Swiss Webster mice infected with *T. gondii* RH strain.

Parameters	Score
Body weight	
5–10% weight loss	1
11–15% weight loss	2
16–20% weight loss	3
>20% weight loss	Euthanasia
Hair condition	
Slight piloerection	2
Prominent piloerection	3
Body temperature	
Up to 2 °C above normal	2
Up to 3 °C above normal	3
Hypothermia (<35 °C)	Euthanasia
Respiratory function	
Tachypnea	2
Dyspnea	3
Pain parameters	
Moderately present orbital tightening	1
Intense present orbital tightening	Euthanasia
Eye discharge	1
Chest breathing	2
Arched posture	3
Environment	
Soft stools or diarrhea	1
Bloody diarrhea	Euthanasia
Behavior	
Tense or nervous during handling	1
In noticeable distress during handling such as tremors, vocalizations, and aggressiveness	3
Specific indicators of infection	
Ascites	Euthanasia

**Table 2:** Concentration of tachyzoites of *T. gondii* RH strain recovered from infected mice (×10^7^ parasites/mL).

Days Post-Infection (DPI)	2 DPI	3 DPI	4 DPI
	1.9	7	1.9
	1.1	3.1	3.3
	2.5	2.6	
	1.9	2.8	
		2.7	
		2.7	
		2.9	
Means value	1.9	3.4	2.6

**Table 3:** Categorical parameters evaluated in Swiss Webster mice infected with *T. gondii* RH strain and euthanized on the 2nd and 3rd DPI.

Clinical/Behavorial Parameters	2nd DPI			3rd DPI		
	No. of Animals	Euthanasia (%)	*p*-Value	No. of Animals	Euthanasia (%)	*p*-Value
Hair Condition						
Normal	13	0	˂0.001	0	0	0.395
Slight piloerection	19	5.3		10	60	
Prominent piloerection	18	83.3		24	79.2	
Respiratory function						
Normal	48	29.2	0.098	26	65.4	0.077
Dyspneia	2	100		8	100	
Moderately present orbital tightening						
No	50	-	NP	29	69	0.293
Yes	0	-		5	100	
Chest breathing						
No	45	26.7	0.031	26	65.4	0.077
Yes	5	80		8	100	
Arched posture						
No	49	30.6	0.32	27	66.7	0.151
Yes	1	100		7	100	
Ascites						
No	37	8.1	˂0.001	14	35.7	˂0.001
Yes	13	100		20	100	

NP: not performed.

**Table 4:** Continuous parameters evaluated in Swiss Webster mice infected with *T. gondii* RH strain and euthanized on the 2nd and 3rd DPI.

Parameters	2nd DPI					3rd DPI				
	No. of Animals	Means	SD	SE	*p*-Value	No. of Animals	Means	SD	SE	*p*-Value
Body weight										
Survivors	34	28.3	3.451	0.592	0.295	9	28.9	1.453	0.484	0.008
Euthanized	16	29.9	5.390	1.347		25	26.2	4.103	0.821	
Body temperature										
Survivors	34	36.9	0.753	0.129	0.241	9	36.9	0.601	0.200	0.446
Euthanized	16	36.6	0.806	0.202		25	37.1	0.702	0.140	

**Table 5:** Multiple linear regression models for clinical and behavioral parameters associated with scores recorded in Swiss Webster mice infected with *T. gondii* RH strain.

Parameters	2nd DPI			3rd DPI		
	Estimate	SE	*p*-Value	Estimate	SE	*p*-Value
Intercept *	35.124	41.402	0.401	−1.8031	10.0918	0.860
Body weight	−0.0263	0.0202	0.200	0.1445	0.0573	0.018
Body temperature	−0.0752	0.1095	0.496	0.0117	0.2597	0.964
Hair condition						
Slight piloerection—Normal	21.508	0.1923	<0.001	-	-	-
Prominent piloerection—Normal	36.839	0.3694	<0.001	-	-	-
Prominent piloerection—Slight piloerection	-	-	-	0.8937	0.3702	0.023
Respiratory function						
Dyspneia—Normal	20.264	0.4008	<0.001	−0.1400	0.5416	0.798
Chest breathing						
Yes—No	16.232	0.3954	<0.001	10.974	0.3882	0.009
Arched posture						
Yes—No	−12.333	0.6466	0.064	0.3686	0.4106	0.378
Ascites						
Yes—No	20.403	0.3593	<0.001	27.711	0.3951	<0.001
Moderately present orbital tightening						
Yes—No	-	-	-	0.4507	0.4759	0.353

* Represents reference level.

## Data Availability

The data that support the findings of this study are available from the corresponding author upon reasonable request.
